# Supplementary material for: Fluoride Concentration in Urine after Supplementation with Quelites in a Population of Adolescents
Source: Foods. 2022 Oct 3;11(19):3071. doi: 10.3390/foods11193071 (PMC9563416; doi:10.3390/foods11193071)
Supplement: Supplementary file 1 [file foods-11-03071-s001.zip › foods-1822837-supplementary.pdf]

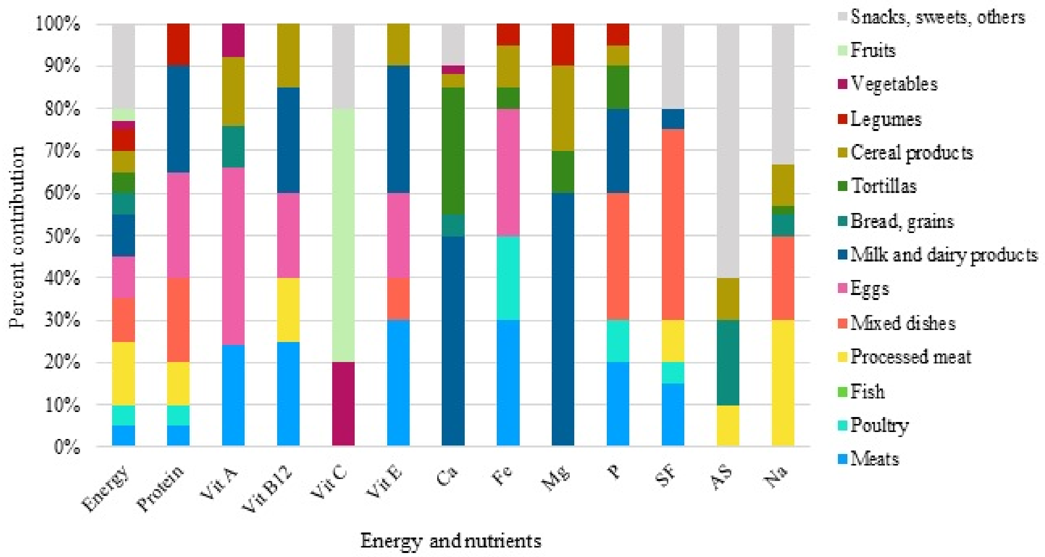

Figure S1. Contribution of food sources in the daily intake of energy, macronutrients, micronutrients and critical nutrients, in participants included in the study. Each color represents a different food source. Vitamin (Vit); Calcium (Ca); Iron (Fe); Magnesium (Mg); phosphorus (P); Sodium (Na); Saturated Fat (SF); Added sugars (AS).

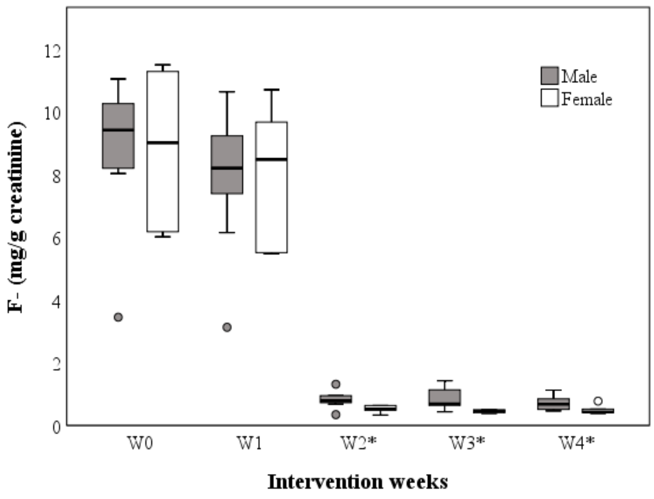

(a)

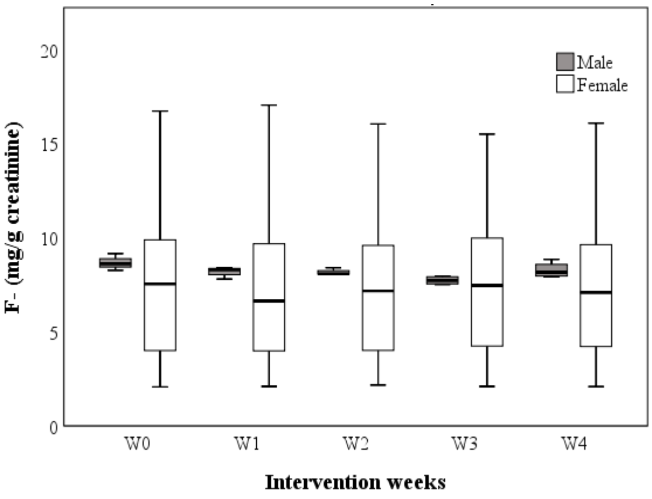

(b)

Figure S2. Comparison of the concentration of F^- (median) by sex. (**a**) Treated group; (**b**) Untreated group. W0: baseline; W1: week 1; W2: week 2; W3: week 3; W4: week 4. *Statistically significant difference by sex according to the Mann-Whitney U test with $p < 0.05$

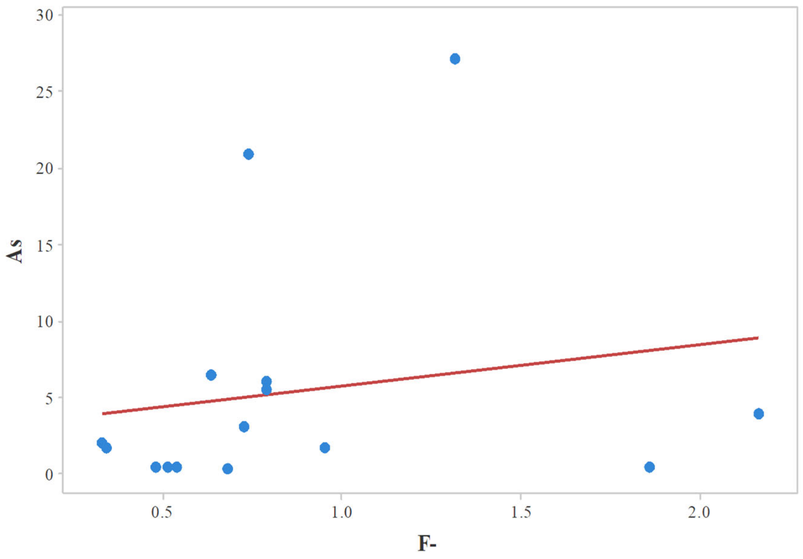

Figure S3. Scatterplot of As concentration (y-axis) vs. concentration of F^- (x-axis) excreted in urine at week 2 of treatment. No correlation was found between the variables.
